# Genome-wide Study on Propofol Requirements: Reply

**DOI:** 10.1097/ALN.0000000000005331

**Published:** 2025-02-04

**Authors:** Sirkku Ahlström, Hanna Granroth-Wilding, Paula Reiterä, Klaus Olkkola, Ritva Jokela, Mari Kaunisto, Eija Kalso

**Affiliations:** 1 University of Helsinki and HUS Helsinki University Hospital, Helsinki, Finland (S.A.). sirkku.ahlstrom@hus.fi

## In Reply:

We would like to thank Drs. Schnider, Egan, and Minto for their comments^1^ on our recent study “Influence of Clinical and Genetic Factors on Propofol Dose Requirements: A Genome-wide Association Study.”^2^ They point out that the drug titration paradox would be particularly relevant in our study, where we titrated propofol to State Entropy, a processed electroencephalograph index, and remifentanil to a hemodynamic endpoint. The main aim of our study was to identify both phenotypic and genetic factors that might be associated with propofol consumption. As the drug titration paradox implies that the “paradoxical” phenomenon that patients with high State Entropy values may have needed higher doses of propofol (or the propofol concentrations have been higher in patients with high Bispectral Index, as in the example by Schnider *et al.*^3^) or that patients with low State Entropy values have used less propofol than expected (if the association were linear), this is exactly the question that we wanted to answer: why is propofol associated with less or more effect in some patients?

Schnider *et al*. suggest that further titration of propofol would have resulted in a greater difference in propofol “consumption” between the most and least sensitive patients and thus strengthened the association between the identified single-nucleotide polymorphisms and propofol consumption (or sensitivity) in our study. We could not agree more, but this would have been quite difficult to achieve in our clinical study where anesthesia consisted not only of propofol but also of remifentanil, nitrous oxide, and rocuronium and where the main goal was to achieve adequate surgical anesthesia.

**Fig. 1.**
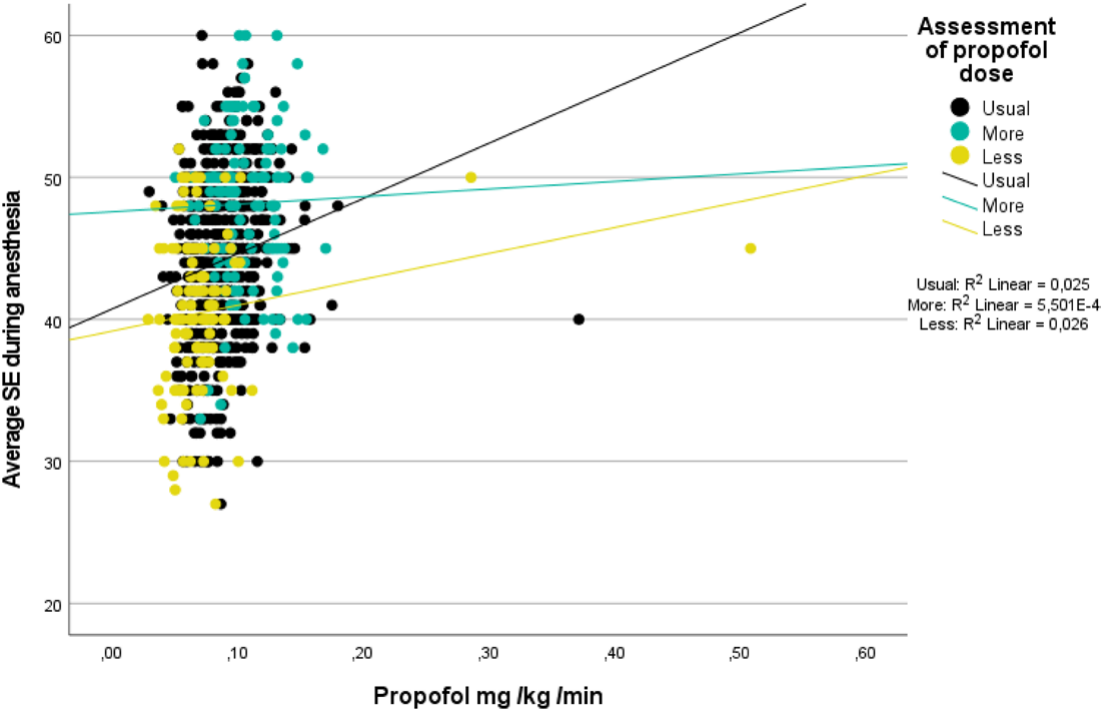
Scatter dot plot of calculated propofol dose values (mg · kg^-1^ · min^-1^) plotted against the values of observed average State Entropy (SE). The subgroups were labeled by the clinician: the patient required more, less, or the usual dose of propofol (infusion rate during surgery). All three groups show SE values, which are scattered between the upper and lower limits of the target area of 45 to 55.

Schnider *et al*. suggested that further exploration of our data, *e.g.*, with a plot of propofol dose *versus* processed electroencephalograph index and remifentanil dose *versus* systolic blood pressure, could provide valuable insights. We have done as they advised and plotted propofol (mg · kg^–1^ · min^–1^) against average State Entropy during anesthesia (fig. 1). Unfortunately, we were not able to plot remifentanil dose *versus* systolic blood pressure due to the way these data were recorded. In addition, remifentanil was not the main topic of our study. Interestingly, we had asked the anesthetists to assess whether they thought that the patient had needed the usual amount of propofol, or more or less than the usual amount. Figure 1 shows that the titration was not entirely “incomplete” because in that case, all yellow dots used to depict the group “less” would have been in the lower left corner of the illustration. The values of *R*^2^ indicate low correlation between State Entropy and the propofol dose. This could highlight the human factor involved in anesthesia: is the clinician satisfied with State Entropy of 55 or should they continue to titrate the infusion to achieve State Entropy at the lower end of the State Entropy limits. Because of this, the State Entropy values reflect not only the patient’s sensitivity to propofol but also the personal “signature” of the anesthetists.

We thank Professor Schnider and his colleagues for their comments, and we agree that the genetic associations might have been stronger had we been able to take the drug titration paradox better into account. We encourage further research on the drug titration paradox and how it should be considered in this type of studies.

## Competing Interests

The authors declare no competing interests.
